# Prevalence of Migraine Disease in Electrohypersensitive Patients

**DOI:** 10.3390/jcm12124092

**Published:** 2023-06-16

**Authors:** Frédéric Greco, Océane Garnier, Valérie Macioce, Marie Christine Picot

**Affiliations:** 1 Department of Anaesthesia and Critical Care Medicine Gui de Chauliac, CHU Montpellier, University of Montpellier, 34295 Montpellier, France; 2 Clinical Research and Epidemiology Unit, CHU Montpellier, University of Montpellier, 34295 Montpellier, France

**Keywords:** migraine, headache, electrohypersensitive, electromagnetic fields, prevalence, multiple chemical sensitivity

## Abstract

Background: The vast majority of electrohypersensitive (EHS) patients present headaches on contact with an electromagnetic source. Clinical features suggest that the headaches of these patients could be a variant of the migraine disease and could be treated as such. We aimed to assess the prevalence of migraine disease in EHS patients using a validated questionnaire. Methods: Patients with EHS defined according to WHO criteria were contacted through EHS patient support associations. They were required to answer a self-questionnaire including clinical data and the extended French version of the ID Migraine questionnaire (ef-ID Migraine) to screen for the migraine disease. Migraine prevalence and its 95% confidence interval (CI) were reported. Patients’ characteristics, symptoms (rheumatology, digestive, cognitive, respiratory, cardiac, mood, cutaneous, headache, perception, genital, tinnitus and tiredness) and impact on daily life were compared between migraineur and non-migraineur patients. Results: A total of 293 patients were included (97% women, mean age 57 ± 12 years). Migraine was diagnosed in 65% (N = 191; 95% CI: 60–71%) with the ef-ID Migraine. The migraine diagnosis was accompanied by nausea/vomiting in 50% of cases, photophobia in 69% or visual disturbances in 38%. All of the 12 symptoms assessed were of higher intensity in migraineurs than in non-migraineurs. The symptoms prevented social life in 88% of migraineurs and 75% of non-migraineurs (*p* < 0.01). Conclusions: Our work encourages us to consider the headaches of these patients as a possible variant of the migraine disease and, possibly, to manage them according to the current recommendations.

## 1. Background

Electrohypersensitivity (EHS), or idiopathic environmental intolerance to electromagnetic fields, is defined by the World Health Organization (WHO) by three criteria: the perception by the subject of various non-specific functional symptoms, the absence of clinical and biological evidence to explain the symptoms and the attribution by the subjects themselves of these symptoms to exposure to electromagnetic fields, which are themselves diverse [1]. EHS affects between 3 and 5% of the French population [2].

The cause and scientific basis of this syndrome are widely debated [3,4,5,6,7]. The relationship between exposure to electromagnetic fields and patients’ symptoms has not yet been formally and reproducibly demonstrated in provocative studies [8,9,10]. However, in their 2020 Joint report, the US National Academies of Medicine, Science and Engineering officially recognized the existence of non-thermal effects of non-ionizing electromagnetic radiation in humans and, in particular, headaches [11]. Moreover, a 2020 study found that the electromagnetic radiation emitted by smartphones was one of the main triggers of migraines in a population of Thai adolescents [12].

One of the symptoms most frequently reported by EHS patients is headaches, which were present in 98% of cases in the largest series reported [13]. The collective expertise report on electromagnetic hypersensitivity of the French national agency for food, environmental and occupational health safety (ANSES) recommends defining whether the headaches of EHS people are in whole or in part migraines and whether these people are more prone to migraines than the rest of the population [2].

Headaches occurring in EHS patients share common features with the migraine disease, such as demographic characteristics of EHS patients, family history, description of the headaches, accompanying signs (photophobia, osmophobia, nausea) and, sometimes, clinical improvement with triptan. This suggests that the headaches of EHS patients could be a variant of the migraine disease and that they could be treated as such.

The aim of this study was to determine the prevalence of the migraine disease using the extended French version of the ID Migraine (ef-ID Migraine) questionnaire in a population of EHS patients [14,15]. Secondary objectives were to compare the characteristics and symptoms of migraineurs versus non-migraineurs.

## 2. Methods

### 2.1. Study Design and Population

The study was validated by the local ethics committee of Montpellier (IRB-MTP_2021_04_202100828 19 April 2021) in accordance with French regulations and registered on the ClinicalTrials.org study registration platform (NCT04845152).

Any French-speaking adult patient meeting the WHO criteria for EHS could participate [1]. The non-inclusion criteria concerned patients refusing to participate.

Patients were informed of the existence of the study through specific French associations of patients with an information letter and a questionnaire available online or by mail by request. Every volunteer could participate from 1 May to 1 December 2021 by answering and sending the completed questionnaire. 

### 2.2. Collected Data

The questionnaire was composed of four parts detailing:

- family history: allergy, food intolerance, asthma, epilepsy, migraines, intolerance to noise, light, smells or vibrations, fibromyalgia, electro-sensitivity and multiple chemical sensitivity (MCS),
- personal history included the same items as well as a history of head trauma and dental care with amalgam placement,
- characteristics of the pathology included the year of the symptoms onset, sources of electromagnetic radiation involved, triggering factor, medical diagnosis, existence of a file at the Departmental House for the Disabled and recognition as a disabled worker,
- EHS symptoms, evaluated from 0 (no symptom) to 10 (very intense and disabling symptoms) in twelve categories. Rheumatology symptoms included pain, cramps and stiffness or weakness of muscles or joints. Digestive symptoms included abdominal pain or cramps, bloating, nausea, diarrhea or constipation. Cognitive symptoms included difficulty in concentrating, memory problems, feeling of disconnection, lack of words or difficulty in making decisions. Respiratory symptoms included irritation of the eyes, shortness of breath, chest tightness or cough. Cardiac symptoms included accelerated or irregular heartbeat, extrasystoles palpitations or discomfort in the chest. Feeling tense or nervousness, irritability, depression, crying or angry outbursts or disinterest in activities that usually motivate were in the mood category. Rash, hives or dry skin concerned cutaneous. Headache symptoms included headaches or feeling of a heavy head or congested face. Perception symptoms included balance disorder or coordination disorder, numbness or tingling in extremities or blurred visual blur. Genital symptoms included pelvic pain or frequent urination. Tinnitus was defined by the sentence “noises in the ears”. Tiredness symptoms corresponded to fatigue or sleep disorders.
- medical treatments ongoing,
- classification of the symptoms’ impact on daily life defined by our clinical experience: stage 1, the symptoms do not modify daily life; stage 2, the symptoms oblige the patient to implement avoidance measures and stage 3, the symptoms prevent a normal social life,
- the extended French version of thew ID Migraine questionnaire. The ef-ID Migraine is a brief, practical and easy-to-use diagnostic tool for migraines. This self-administered questionnaire composed of four items assesses disabling headaches occurring in the past year associated with nausea or vomiting and/or photophobia and/or prodromal visual signs [14,15]. The association should lead to the consideration of a migraine disease, which should be confirmed by a specialized consultation according to the criteria of the international classification of headaches (ICHD-3) [16].

In this study, headache was defined as pain in any region of the head.

The main outcome was the proportion of patients suffering from migraine according to the ef-ID Migraine questionnaire. 

### 2.3. Sample Size

According to our clinical practice, we estimated that 60% of EHS patients seen in consultation suffered from the migraine disease. To estimate this prevalence with an accuracy of ±6% and an alpha error of 5%, 256 patients were required. This sample size was increased by 20% in anticipation of incomplete or unusable questionnaires, yielding a number of subjects to be included that is close to 312.

### 2.4. Statistical Analysis

All the patients included were analyzed. The prevalence of migraine was reported with its 95% confidence interval. For the characteristics of the patients, data were expressed as the number and percentage for qualitative variables. Continuous variables were expressed as the mean and standard deviation when the distribution was Gaussian and as median and quartiles (Q25; Q75) otherwise. Characteristics of migraineurs versus non-migraineurs were compared using the Student or Wilcoxon Mann–Whitney test for continuous variables and the Chi-square or Fisher test for categorical ones.

All analyses were two-tailed, with a *p* value of <0.05 considered statistically significant. The statistical analyses were carried out using SAS^®^ (SAS Institute, Cary, NC, USA).

## 3. Results

During the recruitment period, 317 questionnaires were received, 23 patients were not eligible because they did not report symptoms related to EHS and 1 patient was excluded from the analysis because of an a posteriori refusal to use his data. Thus, 293 patients were analyzed.

Patients’ characteristics are presented in Table 1. They were mainly women, with a mean age of 57 years and a healthy weight. Histories of allergy, food intolerance, multiple chemical sensitivity (MCS) and migraine were present in the majority of patients. All patients reported an onset of symptoms following exposure to electromagnetic radiation, which disappeared at the end of the exposure in 210 patients (81%) and reappeared systematically at a new exposure in 271 patients (97%). EHS-related symptoms were reported a decade earlier. Finally, most patients interviewed reported an impact of EHS on their daily life according to the proposed classification, through a change in the way they live (99%), the implementation of avoidance measures (99%) and/or an impact on social life (83%).

**Figure 1:**
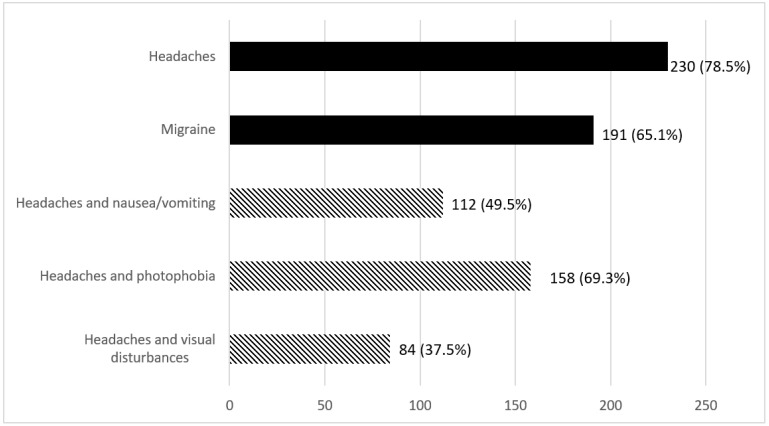
Description of migraine with the ef-ID Migraine tool. Hatched values represent associated symptoms in headaches sufferers. Black values represent all patients studied.

According to the results of the ef-ID Migraine questionnaire presented in Figure 1, 230 (78.5%) patients reported suffering from headaches, of which 191 (83%) could be suspected of having a migraine. The estimated prevalence of migraine was therefore 65% (95% CI 60–71%) in our population of 293 participants. A history of migraine was reported in 142 of the 191 patients identified as migraineurs by the questionnaire (74%) and in 36 of the 102 non-migraineurs (35%). Conversely, 142 of the 178 patients reporting a history of migraine were identified as migraineurs by the questionnaires (80%).

The comparison of patients identified as migraineurs with non-migraineurs is presented in Table 2. Migraine patients were significantly younger, while the gender and BMI were comparable between the two groups.

**Figure 2:**
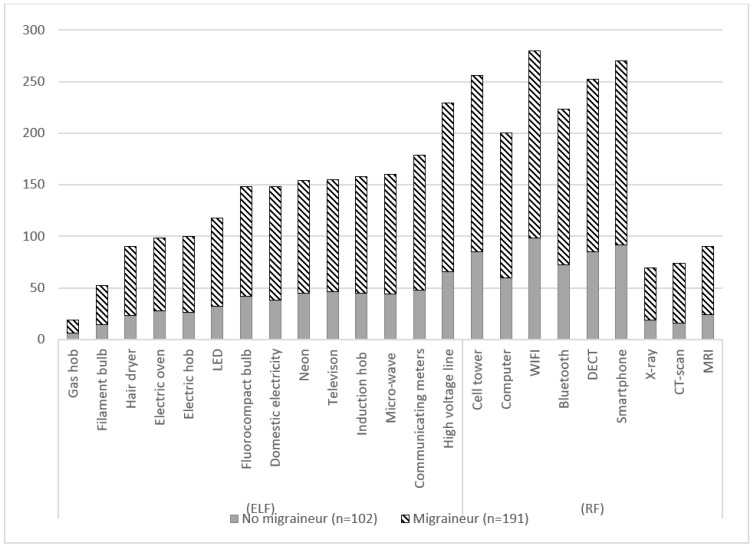
Description of electrohypersensitivity sources expressed according to the number of declarations in each category: all participants, migraineurs identified with the ef-ID-Migraine tool and non-migraineurs. RF: Radio frequencies; ELF: Extremely low frequencies.

Histories of asthma, migraine, fibromyalgia and MCS were more frequent among migraineurs than non-migraineurs. Migraineur patients reported more intolerance to noise, light, vibrations and smells. Furthermore, a greater proportion of migraineurs patients reported discomfort in social life, although the proportion of patients reporting a change in lifestyle or the implementation of avoidance measures was comparable in the two groups. Migraineurs reported more electromagnetic (EM) field sources responsible for EHS symptoms than non-migraineurs (13 ± 5 vs. 10 ± 5, *p* < 0.01). The details of the sources of EM fields are presented in Figure 2.

**Figure 3:**
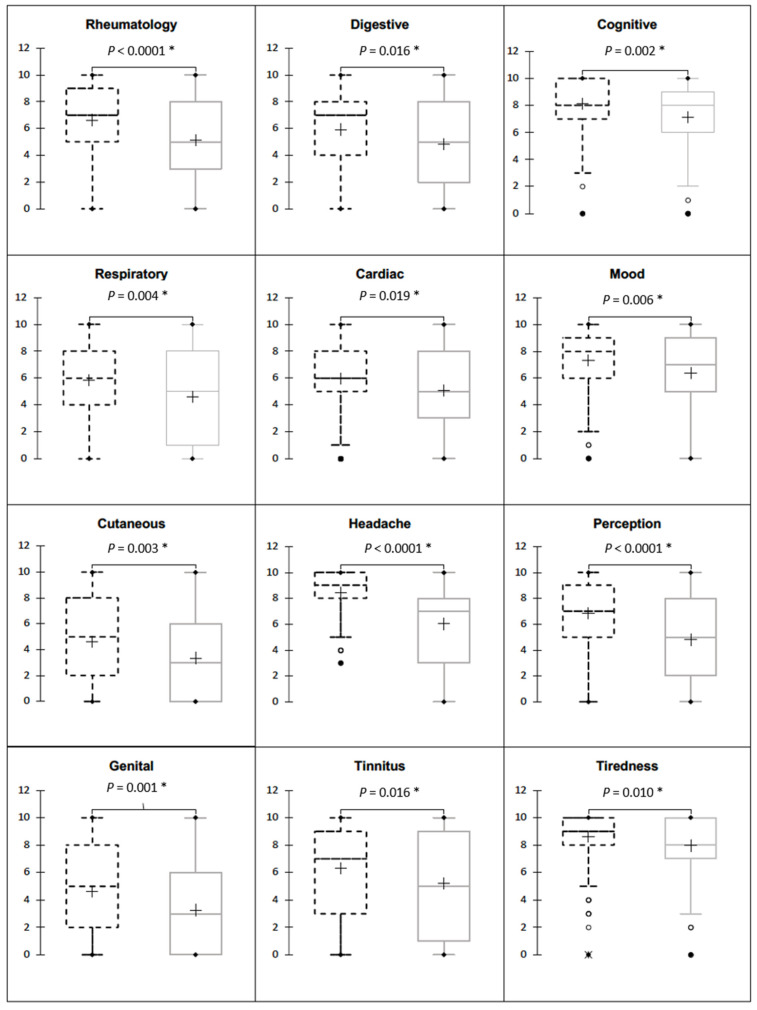
Comparison intensity of the twelve symptoms studied in migraineurs (dashed lines) versus non-migraineurs (grey lines). The cross represents the mean value, the medium line represents the median value, the box represents the first and third quartiles, whiskers represent minimum and maximum adjacent values and circles represent outliers. *: a *p*-value < 0.5 was considered statistically significant.

The total population described symptoms with a median intensity greater than or equal to four out of ten (Figure 3). Tiredness, headache, mood disturbance and cognitive symptoms were the symptoms with intensities greater than or equal to five out of ten in 75% of the patients studied (Table 1).

The comparison of these symptoms in the identified migraineur population versus the non-migraineur population showed a significantly greater intensity in migraine patients (Figure 3). The difference was clinically and statistically relevant for all visceral symptoms. The intensity of symptoms classically attributed to EHS, tiredness, headaches, perceptual disturbances, mood disturbances and cognitive impairment intensity was higher in migraineur patients.

## 4. Discussion

The results of this study show that EHS patients present nonspecific and varied symptoms which meet the current definition of EHS. Similarly, all the patients who responded to the questionnaire reported that symptoms appeared following exposure to electromagnetic fields and disappeared rapidly after exposure for 81% of them and reappeared systematically on re-exposure in almost all patients. Importantly, in 74% of the cases, the diagnosis was made by a physician, particularly by ruling out any other pathology that might explain the clinical signs.

The symptoms affecting the nervous system were the most frequent and the noisiest. In particular, 78.5% of our patients suffered from headaches, whereas their prevalence was estimated at 52% in a literature review [17]. Symptoms were of moderate to severe intensity and seemed to be concomitant with signs of allergy and intolerance to chemical products evocative of a multiple chemical sensitivity, described in 54% of our patients [18]. This observation is relayed in a large study that shows that EHS is associated with MCS patients in 30% of cases and could be a good clinical criterion for the diagnosis of EHS [13].

We observed that the sources involved were mostly related to radiofrequency and that the mean duration of the pathology was about ten years. These observations suggest a symptoms onset in the 2010s, corresponding to the explosion of “wireless” technologies with the generalization of smartphones and WIFI. These findings are in line with the current literature [19]. Of note, these symptoms prevented a normal social life in most patients.

Thus, all these elements make us think that the population studied corresponds well with patients suffering from EHS in accordance with the definitions of the WHO and Belpomme et al.: absence of a known pathology explaining the observed clinical symptoms, association of symptoms with headache, tinnitus, hyperacusis, vertigo, immediate memory loss and attention deficit/concentration, reproducibility of symptoms under the said influence of electromagnetic fields, regression or disappearance of symptoms in the case of said avoidance of electromagnetic fields and association with multiple chemical sensitivity [1,13].

However, the current definition of EHS relies on subjective criteria, which is a source of bias for studies dealing with this population [4]. In order to obtain the most homogeneous population possible to be able to answer our question, we asked the EHS patient associations to help us disseminate the existence of the study, although it has been shown that the patients recruited by this mean had more marked symptoms than people with EHS recruited by a call for participation aimed at the general population [20]. We also note that our sample differs from those in the literature by the preponderance of women, who usually make up about 70% of the subjects, while they represented nearly 97% of our sample [21]. There are additional biases to consider: the number of EHS patients who did not participate in the study cannot be estimated, and each data item studied is derived from the participants’ declarations. In addition, the diagnosis of migraine is not validated by a medical specialist, although the ef-ID-Migraine questionnaire is relatively reliable in the literature [15]. As our study focused on the prevalence of migraine disease in electrosensitive patients, we did not study the characteristics of migraine pathology in depth. Indeed, we have not supplemented the ef-ID Migraine screening questionnaire with more specific questions such as the frequency of headaches, therefore preventing us from determining the proportion of patients suffering from chronic migraine [22]. Nevertheless, our work allowed us to determine that 65% of the interviewed persons were likely to present a migraine disease, with 56% of these patients presenting migraines without aura and 44% presenting migraines with aura.

Taking these reflections into consideration, the important message is that the prevalence of migraine disease identified by the ef-ID-Migraine in our sample was 65% (95% CI 60–71%). This prevalence seems to be much higher than that in the general female French population, where it ranged from 11 to 30% [23,24]. In a Belgian study, the prevalence was 26% in the whole sample and 33.4% in women with the same tool, and among the potential migraineurs, 41% had visual signs, which is close to our findings (44%) [25].

According to the ef-ID-Migraine tool, migraineur patients showed a more pronounced hypersensitivity, with a more frequent intolerance to noise, vibrations, light and odors and symptoms of higher intensity than non-migraineur patients. The results also showed that nearly 61% of the respondents declared a history of migraine. This proportion does not correspond exactly to the proportion identified with the questionnaire (65.2%). It seems that part of the patients studied did not consider themselves as migraine sufferers, and vice versa, highlighting a potential bias in understanding the questions. Patients with a history of migraine and migraineur patients did not take any specific treatment for migraine. Indeed, it is known that migraine sufferers are generally reluctant to take medication. Furthermore, 60% of these patients have elements suggestive of multiple chemical sensitivity; thus, we can assume that they are not taking any treatment because of intolerance and numerous side effects that prevent the benefits. However, it seems that our sample of patients complaining about headaches and probably migraines could at least be offered management of headaches in accordance with the recommendations of the French Society for the Study of Migraines and Headaches [26,27,28].

All these data can suggest a central sensitization syndrome [29]. It would have been interesting to specify this in the course of this investigation, taking the example of our Japanese colleagues who consider a link between migraine, multiple chemical sensitivity and central sensitization syndrome [30]. Regarding migraineurs’ prevalence in this studied population and the link between migraine diseases and central sensitization syndrome, it could be interesting to explore the allodynia phenomenon, related to both diseases but not studied in this survey [31,32,33].

As our work does not specifically investigate the sources of electromagnetic fields most often associated with negative health effects by electrosensitive patients, we can only observe that the impact of electromagnetic fields varies according to the frequency, distance and type of electrical appliance.

This study does not pretend to establish the responsibility of electromagnetic fields in the occurrence of migraines in the patients who answered the questionnaire; however, the fact that electromagnetic fields may be a trigger for migraines is not new. There are many hypotheses for an explanation of headaches in EHS [22,34]. Many migraine patients find that the change in weather is a trigger for their headaches. In the region of Giessen, Germany, Vaitl et al. found a correlation in autumn between sferic activity and the occurrence of migraine attacks [35]. Panangopoulos et al. describe this phenomenon of meteoropathy related to the extremely low frequencies of electromagnetic pulses in thunderstorms and propose a mechanism by which voltage-dependent cationic channels, called electrosensitive, are activated by the polarized and pulsed electromagnetic signal generated by lightning [36]. Moreover, this sensitivity mechanism is also used in tumor reduction or control therapy by administering 27.12 MHz amplitude-modulated radiofrequency electromagnetic fields that are thought to act on cancer cells via certain voltage-dependent calcium channels [37,38]. A review of the literature on the effect of electromagnetic radiation on neuronal ion channels provides insight into the magnitude of the phenomenon and concludes that ion channels represent a major transducer of the effects of electromagnetic fields on the central nervous system [39].

## 5. Conclusions

Our work seems to indicate that the prevalence of migraine disease in EHS individuals is much higher than that in the general population and constitutes the beginning of an answer to the questioning of the French national agency for food environmental and occupational health safety. It incites the continuation of research work and encourages practitioners confronted with electrohypersensitive patients to look for headaches and manage them in accordance with the recommendations of medical societies.

## Figures and Tables

**Table 1:** Characteristics of patients.

Patients’ Description and History	n	Values
Women	292	283 (96.9)
Age, years, mean ± SD	291	56.5 ± 12.3
BMI, kg/m^2^, median [IQ25-75]	292	21.9 [19.7; 24.3]
History of allergy	293	197 (67.2)
History of food intolerance	293	188 (64.2)
History of asthma	293	45 (15.4)
History of seizure	293	10 (3.4)
History of migraine	293	178 (60.7)
History of noise intolerance	293	184 (62.8)
History of light intolerance	293	150 (51.2)
History of vibrations intolerance	293	123 (42,0)
History of smells intolerance	293	185 (63.1)
History of MCS	293	159 (54.3)
History of fibromyalgia	293	61 (20.8)
History of dental care	293	242 (82.6)
History of brain injury	293	60 (20.5)
History of Lyme disease	293	58 (19.8)
Electrohypersensitivity characteristics		
Duration between first EHS symptoms and study, years, median [IQ25-75]	293	10 [5; 16]
Diagnosis of EHS made by a doctor	293	217 (74.1)
Sick leave for EHS	293	64 (21.8)
Medication for EHS	293	97 (33.1)
Departmental House for the Disabled file	293	141 (48.1)
Recognition as a disabled worker	131	94 (71.8)
Trigger identified	293	204 (69.6)
Symptoms appear during exposure to a source of electromagnetic radiation	293	293 (100)
Symptoms stop after exposure is stopped	258	210 (81.4)
Symptoms appear systematically in response to new exposure	280	271 (96.8)
During the past 12 months, symptoms have been occurring more and more frequently	247	157 (63.6)
Symptoms triggered by new sources	220	141 (64.1)
Headaches	293	230 (78.5)
Headaches and nausea/vomiting	226	112 (49.6)
Headaches and photophobia	228	158 (69.3)
Headaches and visual disturbances	224	84 (37.5)
Migraine		191 (65.2)
Migraine with aura		84 (28.7)
Number of electromagnetic field sources identified, mean ± SD	293	12 (±5)
Electrohypersensitivity impact on daily life		
Stage 1: Symptoms do not change the way of living	292	1 (0.3)
Stage 2: Symptoms force putting in place avoidance measures	291	47 (16)
Stage 3: Symptoms prevent social life	290	241 (83)
Intensity of symptoms in EHS patients, median [IQ25-75]		
Rheumatology	287	7 [4–8]
Digestive	284	6 [3–8]
Cognitive	289	8 [7–10]
Respiratory	286	6 [3–8]
Cardiac	290	6 [4–8]
Mood	284	8 [5–9]
Cutaneous	273	4 [0–7]
Headache	287	8 [7–10]
Perception	284	7 [4–8]
Genital	282	4.5 [0–7]
Tinnitus	286	7 [2–9]
Tiredness	286	9 [8–10]

BMI: Body mass index; EHS: Electrohypersensitivity; MCS: Multiple chemical sensibility; IQ: interquartile. Values are n (%) unless otherwise stated.

**Table 2:** Comparison between EHS and migraineur patients identified with the ef-ID Migraine tool and EHS non-migraineurs.

	Migraineur n = 191	Non-Migraineur n = 102	*p*
Age, years, mean ± SD	54.6 ± 11.9	59.97 ± 12.2	<0.01
Women	183 (96.3)	100 (98.0)	0.68
BMI, kg/cm^2^, median (IQ25-75)	21.8 (19.5–24.5)	22.0 (20.1–23.9)	0.55
History of allergy	133 (69.6)	64 (62.7)	0.23
History of food intolerance	130 (68.1)	58 (56.9)	0.06
History of asthma	35 (18.3)	10 (9.8)	0.05
History of seizure	9 (4.7)	1 (0.9)	0.17
History of migraine	142 (74.3)	36 (35.3)	<0.01
History of noise intolerance	130 (68.1)	54 (52.9)	0.01
History of light intolerance	120 (62.8)	30 (29.4)	<0.01
History of vibrations intolerance	90 (47.1)	33 (32.3)	0.01
History of smells intolerance	132 (69.1)	53 (51.9)	<0.01
History of MCS	116 (60.7)	43 (42.2)	<0.01
History of fibromyalgia	49 (25.6)	12 (11.8)	<0.01
History of dental care	162 (84.8)	80 (78.4)	0.17
History of brain injury	45 (23.6)	15 (14.7)	0.07
History of Lyme disease	41 (21.5)	17 (16.7)	0.33
Electrohypersensitivity characteristics			
Symptoms appear during exposure to a source of electromagnetic radiation	191 (100.0)	102 (100.0)	.
Symptoms stop after exposure is stopped	134 (81.2)	76 (81.7)	0.92
Symptoms appear systematically in response to new exposure	178 (97.3)	93 (95.9)	0.50
During the past 12 months, symptoms have been occurring more and more frequently	111 (68.1)	46 (54.7)	0.04
Symptoms triggered by new sources	99 (68.7)	42 (55.3)	0.05
Number of electromagnetic field sources identified, mean ± SD	12.92 ± 4.7	10.33 ± 5.0	<0.01
Electrohypersensitivity impact on daily life			
Symptoms changed the way of living	188 (98.9)	100 (98.0)	0.61
Symptoms forced to put in place avoidance measures	188 (99.8)	99 (97.0)	0.13
Symptoms prevent social life	165 (87.8)	76 (74.5)	<0.01

BMI: Body mass index; EHS: Electrohypersensitivity; MCS: Multiple chemical sensibility; IQ: interquartile. Values are n (%) unless otherwise stated.

## Data Availability

Data will be available upon reasonable request to the corresponding author.

## Abbreviations

EHS: Electrohypersensitive; WHO: World Health Organization; ANSES: Agence nationale de sécurité sanitaire de l’alimentation, de l’environnement et du travail; MCS: Multiple chemical sensitivity; ICDH: International classification of headaches; BMI: Body mass index; EM: Electromagnetic; RF: Radio frequencies; ELF: Extremely low frequencies.
